# Effects of a Sound-Based Meditative Intervention on Anxiety, Depression, Salivary Cortisol Levels, and Blood Pressure: A Randomized Controlled Trial

**DOI:** 10.7759/cureus.115693

**Published:** 2026-09-02

**Authors:** Hemachandran Ravikumar, Suresh Sathyanarayanan

**Affiliations:** 1 Faculty of Medicine, Medical University Pleven, Pleven, BGR; 2 Research, NH Research Park, Chennai, IND; 3 Research and Development, UNS Research Council, Chennai, IND

**Keywords:** cortisol, depression, psychological issues due to stress, sound-based meditation, stress reduction

## Abstract

Background

Psychological stress contributes to mental health symptoms and cardiovascular risk through pathways involving anxiety, depression, cortisol secretion, autonomic dysregulation, and elevated blood pressure. Sound-based meditation is a nonpharmacological approach that may support stress management, but randomized controlled trial evidence remains limited.

Objective

The objective was to evaluate whether a structured sound-based meditation intervention was associated with lower post-intervention anxiety symptoms, measured using the Hospital Anxiety and Depression Scale-Anxiety subscale (HADS-A), than a remotely delivered music-control condition after four weeks. Depressive symptoms, salivary cortisol concentration, systolic blood pressure, and exploratory diastolic blood pressure were also assessed.

Methodology

This parallel-group randomized controlled trial enrolled 100 adults aged 21-65 years from meditation centers and surrounding communities in Tamil Nadu, India. Participants were randomly assigned in a 1:1 ratio to a sound-based meditation intervention group (*n* = 50) or a remotely delivered music-control group (*n* = 50). The intervention group attended supervised, face-to-face, 60-minute daily sound-based meditation sessions for four weeks, involving recorded 528-Hz audio and acoustic instruments, including singing bowls, gongs, bells, and tingshas. The control group attended supervised 60-minute Google Meet sessions using two standardized, licensed music tracks with scheduled silent intervals. The primary analysis used analysis of covariance (ANCOVA) to compare post-intervention HADS-A scores between groups, adjusted for baseline HADS-A scores. Corresponding ANCOVA models evaluated HADS-D, salivary cortisol, and systolic blood pressure as secondary outcomes; diastolic blood pressure was exploratory. Holm adjustment was applied across the five reported outcomes.

Results

Observed baseline characteristics were comparable between groups. In baseline-adjusted ANCOVA analyses, post-intervention HADS-A scores were lower in the intervention group than in the music-control group (adjusted mean difference = -2.55, 95% confidence interval (CI): -3.62 to -1.48; Holm-adjusted *P* < 0.001; partial *η*^2^ = 0.19). Corresponding adjusted differences favored the intervention group for HADS-D (adjusted mean difference = -2.39, 95% CI: -3.36 to -1.43; Holm-adjusted *P* < 0.001; partial *η*^2^ = 0.20), salivary cortisol (-2.54 nmol/L, 95% CI: -2.95 to -2.13; Holm-adjusted *P* < 0.001; partial *η*^2^ = 0.61), systolic blood pressure (-7.62 mmHg, 95% CI: -9.36 to -5.89; Holm-adjusted *P* < 0.001; partial *η*^2^ = 0.44), and exploratory diastolic blood pressure (-2.22 mmHg, 95% CI: -3.00 to -1.43; Holm-adjusted *P* < 0.001; partial *η*^2^ = 0.24). No adverse events were reported.

Conclusions

This randomized controlled trial provides preliminary evidence that participation in a structured sound-based meditation program was associated with greater short-term improvements in anxiety, depression, salivary cortisol, systolic blood pressure, and exploratory diastolic blood pressure compared with a remotely delivered music-control condition. These findings should be confirmed in larger, fully standardized randomized controlled trials with longer follow-up and diverse populations.

## Introduction

Psychological stress is a major public health problem and makes a significant contribution to the development of mental health disorders, cardiovascular disease, and impaired quality of life [1]. Chronic activation of stress-response systems has been linked to increased cortisol secretion, autonomic nervous system dysregulation, hypertension, anxiety symptoms, and depressive symptomatology [1,2]. This has prompted greater interest in complementary and integrative approaches that may help alleviate stress-related symptoms and enhance overall well-being. Among such approaches, meditation-based interventions have received significant scientific attention because of their potential to affect both psychological and physiological stress responses [3,4].

Meditation is a mind-body practice involving the intentional regulation of attention and awareness to promote mental calmness and present-moment awareness. In sound-based meditation, auditory stimuli such as structured tones, rhythmic sounds, or specific sound frequencies serve as a focal point for attention, helping participants maintain concentration, reduce distracting thoughts, and facilitate a relaxed meditative state. Meditation has been associated with reductions in anxiety, depression, perceived stress, and cardiovascular risk factors [3,4]. However, meditation practices are quite heterogeneous, and there is an important need to evaluate specific interventions with rigorous research designs. In the past decade, the evidence supporting meditation-based interventions has grown dramatically. A large systematic review and meta-analysis by Goyal et al. reported that meditation programs were associated with improvements in anxiety, depression, and psychological stress when compared with active and passive control conditions [3].

Goldberg et al. similarly found that mindfulness-based interventions were associated with improvements in psychiatric symptoms, including anxiety and depression [4]. These findings indicate that meditation-based practices may confer significant psychological benefits and warrant assessment in randomized controlled trial settings. Sound-based meditation is based on structured auditory stimulation using recorded sound exposure and acoustic instruments such as singing bowls, bells, gongs, and tingshas [5-7]. These interventions are increasingly used in wellness and integrative medicine settings and are believed to promote relaxation, attentional focusing, emotional regulation, and increased self-awareness. Previous studies have reported improvements in mood, perceived stress, and physiological markers of relaxation following meditation-based and sound-based interventions, but the evidence is limited by small sample sizes, nonrandomized designs, poor control conditions, and inconsistent reporting of physiological outcomes [5-9].

Physiological markers of stress have also been an increasing area of focus in addition to psychological outcomes. Systematic reviews have indicated that mindfulness and other mind-body interventions may affect biological stress pathways, including hypothalamic-pituitary-adrenal (HPA) axis activity and cortisol regulation [8]. Meta-analyses suggest that meditation-based interventions may reduce blood pressure and improve cardiovascular regulation [9]. In addition, de Witte et al. reported reductions in stress-related outcomes following music-based interventions, supporting the potential role of structured auditory experiences in stress management [5].

However, few randomized controlled trials have examined both psychological and physiological outcomes in structured sound-based meditation interventions [5-9]. The current study examined a structured sound-based meditation intervention using a randomized controlled design, with psychological and physiological outcome measures and supervised adherence monitoring during the intervention period. The primary objective was to evaluate reductions in stress, tension, anxiety, and depressive symptoms after the intervention period. The trial registration specified the Hospital Anxiety and Depression Scale (HADS) and the Profile of Mood States-Short Form (POMS-SF) as primary psychological outcome measures. In this report, the available HADS-A and HADS-D data are presented; POMS-SF data are not reported. Salivary cortisol concentration, systolic blood pressure, and diastolic blood pressure were evaluated as physiological outcomes, with diastolic blood pressure reported as exploratory.

## Materials and methods

Methodology

Study Design

A parallel-group randomized controlled trial design was used in this study to examine the effects of a structured sound-based meditation intervention on anxiety, depression, salivary cortisol, systolic blood pressure, and diastolic blood pressure. The study was conducted according to the ethical principles of the Declaration of Helsinki and CONSORT recommendations for randomized controlled trials [10]. The study was ethically approved by the Institutional Ethics Committee (Approval ID: UNS/IEC/2024/12/11). All study participants provided written informed consent before enrollment. The trial was prospectively registered with the Clinical Trials Registry of India (CTRI; CTRI/2025/01/078996).

Study Setting

The study was carried out in Tamil Nadu, India, at meditation and wellness centers between January 2025 and April 2025. The intervention group attended supervised in-person meditation sessions at the study center, while the control group attended remotely via a supervised online platform. All assessments, intervention sessions, and follow-up procedures were conducted according to a prespecified study protocol.

Participants

Recruitment: Participants were recruited from meditation centers and surrounding communities through announcements, referrals, and voluntary participation. A total of 100 participants meeting the eligibility criteria were enrolled in the study.

Inclusion criteria: Inclusion criteria were age between 21 and 65 years, provision of written informed consent, willingness to participate in daily intervention sessions for four weeks, physical capability to participate in meditation sessions, ability to complete the 14-item HADS and undergo the required physiological assessments, and willingness to adhere to the study procedures.

Exclusion criteria: Participants were excluded if they had severe psychiatric disorders requiring immediate clinical intervention, uncontrolled cardiovascular disease, significant hearing impairment, inability to complete baseline or follow-up assessments, or were currently participating in another behavioral intervention study.

Sample size: A total of 100 participants were recruited and randomly allocated to intervention and control groups (50 participants per group). The sample size was determined based on feasibility, participant availability, and the exploratory nature of the study.

Randomization

Randomization was carried out after completion of baseline assessments, using a 1:1 allocation ratio. In accordance with the CTRI registration, allocation was undertaken using a lottery-based procedure, and case record numbers were used for allocation concealment. Participants were blinded to their group allocation. Participants were assigned to the intervention group (*n* = 50, 50%) or the control group (*n* = 50, 50%). Baseline demographic and clinical characteristics were compared to assess group comparability. Age, sex distribution, HADS anxiety score, HADS depression score, salivary cortisol level, systolic blood pressure, and diastolic blood pressure did not differ significantly between groups (all *P* > 0.05).

Intervention Group

Participants in the intervention group participated in mandatory face-to-face group meditation sessions at the study center. The intervention involved daily 60-minute sessions for four consecutive weeks. Each session included recorded sounds, including 528-Hz audio, and acoustic instruments such as singing bowls, gongs, bells, and tingshas. Sessions lasted 60 continuous minutes, without scheduled silent or rest intervals between successive auditory components. The recorded sound constituted the principal component of the intervention, while the acoustic instruments were used as complementary auditory elements. No formal musical score was used. No spoken guidance or standardized verbal meditative script was provided. Before each session, participants were instructed to remain in a comfortable seated or lying position and focus on the auditory experience. Sound was delivered through the same 5.1-channel surround-sound system at a standardized, comfortable listening level acceptable to all participants, and this setting was maintained consistently during the sessions. Sound-pressure levels were not objectively recorded in decibels. The intervention was delivered by three trained meditation instructors under the direct supervision of a multidisciplinary team comprising four psychologists. The instructors facilitated the sound-based meditation program, while the psychologists monitored participant well-being, engagement, and adherence. All participants followed the same structured intervention procedure throughout the four-week study period. In addition to the supervised center-based sessions, participants were advised to undertake 40 minutes of home-based self-meditation daily using recorded 528-Hz sound material, divided into 20 minutes in the morning and 20 minutes at night. Therefore, the documented intervention dose comprised a 60-minute supervised center-based session and advised 40 minutes of home practice each day. Attendance and home-practice completion were recorded.

Control Group

Participants in the control group received a remotely delivered music-control intervention via an internal Google Meet platform. The control condition was designed to control for time commitment, participant attention, environmental engagement, and study participation but not the active components of the sound-based meditation intervention. Participants were asked to attend each scheduled session using laptop speakers, external speakers, or Bluetooth speakers, whichever was available and preferred. Participants were asked to set the audio level at a comfortable listening volume that allowed for clear perception of the audio without discomfort or distraction. All control participants received the same audio content at the same time through the Google Meet platform.

The control intervention consisted of two licensed Epidemic Sound music tracks: *Metamorphosis* by Laura Platt (4 minutes 13 seconds) and *Sensai Masopi Sky* by DEX 1200 (2 minutes 12 seconds). The tracks did not contain the recorded 528-Hz audio, singing bowls, gongs, bells, tingshas, or other acoustic components used in the intervention group. Each control cycle consisted of *Metamorphosis*, followed by three minutes of silence, then *Sensai Masopi Sky*, followed by a further three minutes of silence, with a total cycle duration of 12 minutes and 25 seconds. This cycle was repeated four times. The remaining session time consisted of a final playback of *Metamorphosis*, three minutes of silence, a playback of *Sensai Masopi Sky*, and 55 seconds of silence, completing the 60-minute session. Both *Metamorphosis* and *Sensai Masopi Sky* are fully licensed for unlimited use in the production, and permission to use and reproduce the tracks has been obtained from the copyright owners.

Attendance was required. Participants were asked to keep their video cameras on during the entire session, remain visible to study personnel during the intervention period, and stay connected until the session ended. Attendance was constantly monitored and recorded. The control group sessions were supervised by three appointed supervisors and members of the psychology team. Supervisors monitored participants’ attendance, engagement, and compliance with study procedures on an ongoing basis via the videoconferencing platform.

Intervention Fidelity and Adherence

Several procedures were implemented to maintain intervention fidelity and participant adherence. All sessions in the intervention group were of equal length, delivered using the same procedure, and were directly supervised by meditation instructors and psychologists. The intervention group received the same structured 60-minute sound-based session each day, while the control group received the standardized two-track music schedule with the prespecified silent intervals described above. Attendance for the control group was monitored continuously through Google Meet, and attendance records were maintained for each session. Attendance was mandatory in both groups, and attendance records were maintained for each scheduled session. All 100 randomized participants completed the post-intervention assessment and were included in the final analysis. Among intervention-group participants, home-practice records showed 1,272 completed home-practice entries and 128 missed entries out of 1,400 possible participant-days.

Outcome Measures

The trial was prospectively registered, with HADS and the Profile of Mood States-Short Form (POMS-SF) identified as psychological outcome measures. In the present report, the primary analysis examined the between-group difference in change in HADS-A score from baseline to the end of the four-week intervention. Secondary outcomes were the corresponding between-group changes in HADS-D score, salivary cortisol concentration, and systolic blood pressure. Diastolic blood pressure was also recorded and is reported as an exploratory physiological outcome. POMS-SF data were not available for inclusion in the final analysis dataset.

Hospital Anxiety and Depression Scale

Symptoms of anxiety and depression were assessed using the HADS [11], a validated 14-item self-report measure comprising seven items for anxiety (HADS-A) and seven items for depression (HADS-D). Each item is scored from 0 to 3, resulting in a possible score of 0-21 for each subscale. Scores of 0-7 indicate a normal range, 8-10 indicate a borderline abnormal range, and 11-21 indicate an abnormal range. The anxiety and depression subscales were scored independently, with higher scores indicating greater symptom severity. Assessments were performed at baseline and immediately after the four-week intervention period. HADS-A and HADS-D scores were calculated independently as the sum of their respective seven-item scores, and baseline and post-intervention scores were available for all participants in the final analysis dataset.

Salivary Cortisol

Salivary cortisol was used as a physiological stress response and as a biomarker of the HPA axis [12,13]. Salivary cortisol concentrations were reported in nmol/L. All saliva samples were collected during a standardized morning assessment window to reduce diurnal variation in cortisol secretion and under similar pre- and post-intervention conditions. Participants were requested to abstain from eating, drinking caffeinated beverages, alcohol consumption, smoking, and vigorous physical activity before sampling. Samples were obtained with the Salivette® cortisol collection device (Sarstedt, Nümbrecht, Germany). Samples were stored at -20 °C within four hours of collection until analysis. Samples were thawed and centrifuged before laboratory analysis to remove mucins and cellular debris. Salivary cortisol levels were measured with a commercially available enzyme-linked immunosorbent assay (ELISA) kit in accordance with the manufacturer's instructions. Standardized collection procedures were used to obtain baseline and post-intervention measures of cortisol to evaluate changes in physiological stress response following the intervention.

Blood Pressure Assessment

Systolic and diastolic blood pressure were measured using an Omron HEM-7120 Fully Automatic Digital Blood Pressure Monitor (Omron Healthcare Co., Kyoto, Japan) with Intellisense™ technology and an adult cuff for arm circumferences of 22-32 cm. Before assessment, participants were comfortably seated with back support and feet flat on the floor. Participants were allowed 5 minutes of quiet rest before blood pressure measurement. At each assessment session, two blood pressure readings were obtained, and the average of the two readings was used for statistical analysis [14]. Blood pressure measurements were obtained at baseline and at the end of the intervention period under standardized conditions. Systolic blood pressure was analyzed as a secondary outcome, while diastolic blood pressure was reported as an exploratory physiological outcome.

Outcome assessors responsible for blood pressure measurement and laboratory personnel responsible for salivary cortisol analysis were blinded to participant group allocation. HADS questionnaires were completed by participants, and scoring was performed using the standard HADS scoring procedure without disclosure of group allocation to the outcome assessment team.

Adverse Events

Participants were monitored throughout the intervention period for adverse events or harms. No intervention-related adverse events or harms were reported during the study period.

Statistical Analysis

Data were analyzed using Python version 3.12.13 (Python Software Foundation, Wilmington, DE), with pandas version 2.2.3, SciPy version 1.17.0, and NumPy version 2.3.5 (NumFOCUS, Austin, TX). Continuous variables are presented as mean ± standard deviation (SD), and categorical variables are presented as frequencies and percentages. Baseline comparability between groups was assessed using independent-samples t-tests for continuous variables and chi-square tests for categorical variables, as appropriate. Normality of outcome distributions was assessed using the Shapiro-Wilk test. Paired-samples t-tests were used to describe within-group changes from baseline to post-intervention.

The primary analysis evaluated the between-group effect on post-intervention HADS-A score using analysis of covariance (ANCOVA), with treatment group as the independent variable and baseline HADS-A score as a covariate. Corresponding ANCOVA models were used for HADS-D score, salivary cortisol concentration, and systolic blood pressure as secondary outcomes. Diastolic blood pressure was analyzed as an exploratory physiological outcome. Adjusted mean differences, 95% confidence intervals (CIs), and two-sided *P*-values were calculated. To account for multiple outcome testing, *P*-values were adjusted using the Holm procedure.

For descriptive sensitivity analyses, change scores were calculated as post-intervention value minus baseline value, with negative values indicating a reduction from baseline. Independent-samples t-tests were used to compare change scores between groups. Cohen's *d* effect sizes were calculated for the change-score comparisons. No outcome data were missing in the final analysis dataset. Statistical significance was defined as a two-sided *P*-value less than 0.05.

## Results

Participant flow

**Figure 1:**
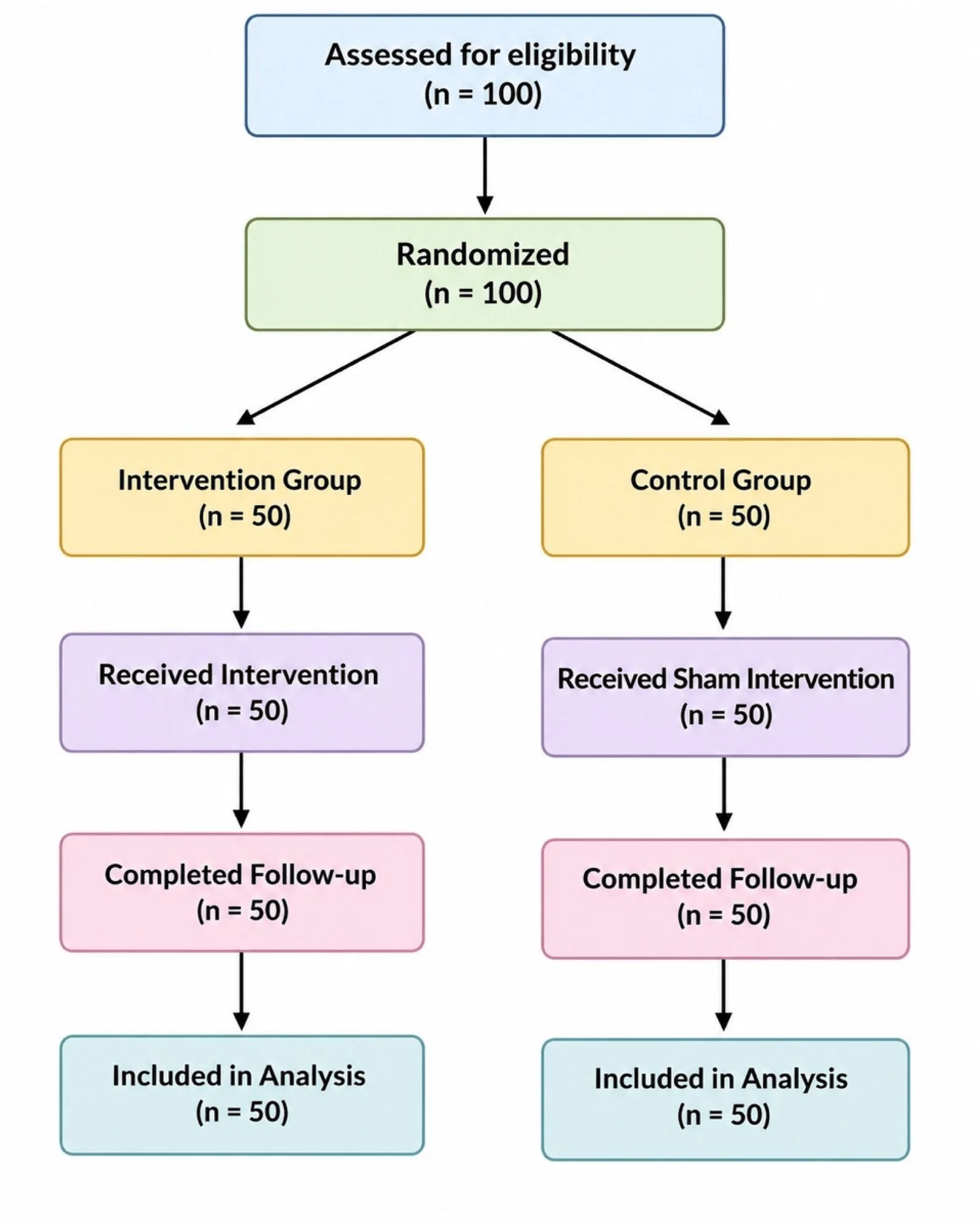
Participant flow through the study. No participants were lost to follow-up, discontinued the intervention, or were excluded from the final analysis.

A total of 100 participants were screened for eligibility and enrolled in the study. Following the baseline assessment, participants were randomly assigned to either the sound-based meditation intervention group (*n* = 50) or the music-control condition group (*n* = 50). All participants completed the intervention period and the follow-up assessments. There were no withdrawals from the study and no missing outcome data. Thus, all randomized participants were included in the final analyses. The complete participant flow is presented in Figure 1.

Baseline characteristics

Table 1 presents the demographic and available baseline clinical characteristics of the participants. No statistically significant between-group differences were observed at baseline.

**Table 1:** Baseline characteristics of participants. Values are presented as mean ± standard deviation or number of participants. Continuous variables were compared using two-sided independent-samples t-tests, and sex distribution was compared using Pearson's chi-square test. Diastolic blood pressure was recorded as an exploratory physiological measure. SD, standard deviation

Variable	Control (*n* = 50)	Intervention (*n* = 50)	Test statistic	*P*-value
Age (years)	45.92 ± 14.43	46.82 ± 12.64	*t*(98) = -0.33	0.741
Sex, female/male	25/25	25/25	*χ*²(1) = 0.00	1.000
Hospital Anxiety and Depression Scale-Anxiety (HADS-A)	9.92 ± 2.83	10.68 ± 2.65	*t*(98) = -1.39	0.169
Hospital Anxiety and Depression Scale-Depression (HADS-D)	10.48 ± 3.16	9.90 ± 3.25	*t*(98) = 0.90	0.368
Salivary cortisol (nmol/L)	15.18 ± 1.30	15.20 ± 1.29	*t*(98) = -0.08	0.939
Systolic blood pressure (mmHg)	130.12 ± 5.50	130.08 ± 5.17	*t*(98) = 0.04	0.970
Diastolic blood pressure (mmHg)	80.90 ± 2.64	80.24 ± 2.97	*t*(98) = 1.17	0.244

Baseline comparability

There were no statistically significant differences in demographic characteristics or available outcome measures between groups at baseline (all *P* > 0.05), indicating comparable observed baseline characteristics before the intervention.

Descriptive within-group changes from baseline to post-intervention are shown in Table 2. The results are presented for descriptive purposes only, and the primary analysis of the randomized controlled trial is presented in Table 3.

**Table 2:** Within-group changes. Each HADS subscale ranges from 0 to 21, with higher scores indicating greater symptom severity; scores of 0-7 indicate the normal range, 8-10 the borderline abnormal range, and 11-21 the abnormal range. Change scores were calculated as post-intervention value minus baseline value. Negative values indicate reductions from baseline. Results are presented for descriptive purposes only; the primary baseline-adjusted between-group analysis is presented in Table 3. Diastolic blood pressure was analyzed as an exploratory physiological outcome. HADS-A, Hospital Anxiety and Depression Scale-Anxiety; HADS-D, Hospital Anxiety and Depression Scale-Depression; BP, blood pressure

Outcome	Group	Baseline mean	Post-intervention mean	Mean change
HADS-A	Control	9.92	10.32	+0.40
	Intervention	10.68	7.68	-3.00
HADS-D	Control	10.48	9.80	-0.68
	Intervention	9.90	7.40	-2.50
Salivary cortisol (nmol/L)	Control	15.18	14.76	-0.42
	Intervention	15.20	12.22	-2.98
Systolic BP (mmHg)	Control	130.12	128.22	-1.90
	Intervention	130.08	120.60	-9.48
Diastolic BP (mmHg)	Control	80.90	80.54	-0.36
	Intervention	80.24	77.92	-2.32

**Table 3:** Baseline-adjusted between-group differences in post-intervention outcomes. Results were obtained using analysis of covariance (ANCOVA), with post-intervention outcome as the dependent variable, study group as the independent variable, and the corresponding baseline outcome as a covariate. *P*-values were adjusted using the Holm procedure across the five reported outcomes. Negative adjusted differences indicate lower post-intervention values in the intervention group after adjustment for baseline values. Partial η^2^ is the ANCOVA effect size. Each HADS subscale has a possible score range of 0-21. Scores of 0-7 indicate a normal range, 8-10 indicate a borderline abnormal range, and 11-21 indicate an abnormal range. Higher scores indicate greater symptom severity. Diastolic blood pressure was analyzed as an exploratory outcome. HADS-A, Hospital Anxiety and Depression Scale-Anxiety; HADS-D, Hospital Anxiety and Depression Scale-Depression; BP, blood pressure; CI, confidence interval

Outcome	Adjusted difference (Intervention - Control)	95% CI	Test statistic	Holm-adjusted *P*-value	Partial *η*^2^
HADS-Anxiety	-2.55	-3.62 to -1.48	*t*(97) = -4.73	<0.001	0.19
HADS-Depression	-2.39	-3.36 to -1.43	*t*(97) = -4.93	<0.001	0.20
Salivary cortisol (nmol/L)	-2.54	-2.95 to -2.13	*t*(97) = -12.38	<0.001	0.61
Systolic BP (mmHg)	-7.62	-9.36 to -5.89	*t*(97) = -8.72	<0.001	0.44
Diastolic BP (mmHg)	-2.22	-3.00 to -1.43	*t*(97) = -5.61	<0.001	0.24

Descriptive findings

The intervention group showed descriptive reductions from baseline in HADS-A, HADS-D, salivary cortisol, systolic blood pressure, and diastolic blood pressure. The control group showed smaller reductions in HADS-D, salivary cortisol, systolic blood pressure, and diastolic blood pressure, while HADS-A increased slightly. The primary baseline-adjusted between-group analysis is presented in Table 3.

Baseline-adjusted between-group analysis

Table 3 presents baseline-adjusted between-group comparisons using ANCOVA. HADS-A was the primary analysis in this report; HADS-D, salivary cortisol, and systolic blood pressure were secondary analyses, while diastolic blood pressure was exploratory.

The magnitude and clinical interpretation of the effect sizes for all study outcomes are summarized in Table 4.

**Table 4:** Interpretation of ANCOVA effect sizes. Effect sizes were calculated as partial eta squared (partial *η*^2^) from the baseline-adjusted ANCOVA models. Using conventional benchmarks, values of approximately 0.01, 0.06, and 0.14 represent small, medium, and large effects, respectively. Diastolic blood pressure was analyzed as an exploratory outcome. HADS-A, Hospital Anxiety and Depression Scale-Anxiety; HADS-D, Hospital Anxiety and Depression Scale-Depression; BP, blood pressure; ANCOVA, analysis of covariance

Outcome	Partial *η*^2^	Statistical interpretation
HADS-A	0.19	Large
HADS-D	0.20	Large
Salivary cortisol (nmol/L)	0.61	Large
Systolic BP (mmHg)	0.44	Large
Diastolic BP (mmHg)	0.24	Large

Anxiety outcomes

Consistent with the baseline-adjusted ANCOVA analysis presented in Table 3, participants in the sound-based meditation intervention group had significantly lower post-intervention HADS-A scores than participants in the music control group after adjustment for baseline HADS-A score. The adjusted between-group difference was -2.55 points (95% CI: -3.62 to -1.48; Holm-adjusted *P* < 0.001), with a large adjusted effect size (partial *η*^2^ = 0.19).

Depression outcomes

Consistent with Table 3, the intervention group had significantly lower post-intervention HADS-D scores than the control group after adjustment for baseline HADS-D score. The adjusted between-group difference was -2.39 points (95% CI: -3.36 to -1.43; Holm-adjusted *P* < 0.001), with a large adjusted effect size (partial *η*^2^ = 0.20).

Salivary cortisol outcomes

As presented in Table 3, participants receiving the sound-based meditation intervention had significantly lower post-intervention salivary cortisol concentrations than participants in the music control group after adjustment for baseline cortisol concentration. The adjusted between-group difference was -2.54 nmol/L (95% CI: -2.95 to -2.13; Holm-adjusted *P* < 0.001), with a large adjusted effect size (partial *η*^2^ = 0.61).

Blood pressure outcomes

As shown in Table 3, participants who received the sound-based meditation intervention had significantly lower post-intervention systolic blood pressure than control-group participants after adjustment for baseline systolic blood pressure. The adjusted between-group difference was -7.62 mmHg (95% CI: -9.36 to -5.89; Holm-adjusted *P* < 0.001), with a large adjusted effect size (partial *η*^2^ = 0.44).

Diastolic blood pressure, analyzed as an exploratory outcome, was also lower in the intervention group after adjustment for baseline diastolic blood pressure. The adjusted between-group difference was -2.22 mmHg (95% CI: -3.00 to -1.43; Holm-adjusted *P* < 0.001), with a large adjusted effect size (partial *η*^2^ = 0.24).

Participant adherence

Attendance was mandatory in both study groups. Participants assigned to the intervention group attended supervised in-person sessions delivered by trained meditation instructors and psychologists. Participants assigned to the control group attended supervised online sessions through Google Meet and remained visible throughout each session with video enabled. Attendance was recorded throughout the intervention period. All 100 randomized participants completed the post-intervention assessment and were included in the final analysis. Among intervention-group participants, home-practice records showed 1,272 completed entries and 128 missed entries out of 1,400 possible participant-days.

Summary of findings

Compared with the music control condition, the structured sound-based meditation intervention was associated with lower post-intervention anxiety, depression, salivary cortisol, systolic blood pressure, and exploratory diastolic blood pressure after adjustment for baseline values. The largest adjusted effect size was observed for salivary cortisol, followed by systolic blood pressure, diastolic blood pressure, HADS-D, and HADS-A. The descriptive changes in Table 2, baseline-adjusted ANCOVA findings in Table 3, and effect-size summary in Table 4 support the potential value of the intervention; however, the findings should be interpreted in light of the study limitations.

## Discussion

This randomized controlled trial examined the association of a structured sound-based meditation program with anxiety, depression, salivary cortisol, systolic blood pressure, and exploratory diastolic blood pressure. In the baseline-adjusted ANCOVA analyses, participants in the intervention group had lower post-intervention HADS-A, HADS-D, salivary cortisol, systolic blood pressure, and diastolic blood pressure values than participants in the music control group. HADS-A was the primary analysis in this report, while HADS-D, salivary cortisol, and systolic blood pressure were secondary analyses; diastolic blood pressure was exploratory. The groups had comparable observed baseline characteristics, all participants completed post-intervention assessments, and no adverse events were reported. All reported ANCOVA effect sizes were large according to conventional partial eta squared benchmarks; however, statistical effect size should not be interpreted as evidence of clinical significance.

Our psychological findings are broadly consistent with previous research on meditation. Goyal et al. [3] reported improvements in anxiety, depression, and psychological stress following meditation programs. Goldberg et al. [4] also found that mindfulness-based interventions may reduce symptoms across several psychiatric conditions. The present findings add to this evidence by suggesting that meditation supported by structured sound exposure may be associated with lower anxiety and depressive symptom scores. Comparisons with earlier studies should be made cautiously because study populations, intervention procedures, outcome measures, and comparator conditions differed.

Our findings are also consistent with earlier work on music- and sound-based practices. De Witte et al. [5] found that music interventions were associated with lower stress-related outcomes. Goldsby et al. [6] reported improvements in tension, mood, and well-being after singing-bowl sound meditation, while Landry [7] observed psychological and physiological changes associated with Himalayan singing-bowl meditation. The present study used randomized group allocation, supervised daily sessions, a music control condition, and both psychological and physiological measurements. However, the intervention and control conditions differed in both sound exposure and delivery setting; therefore, the independent effect of sound-based meditation cannot be isolated fully.

The reductions in salivary cortisol and blood pressure are consistent with literature suggesting that meditation and mindfulness practices may influence stress-related physiological responses [8,9]. Lower diastolic blood pressure was also observed as an exploratory finding. These results are encouraging, but the biological mechanisms responsible for the observed changes were not directly assessed.

Focused listening, relaxation, emotional regulation, and continued meditative engagement may have contributed to the observed findings [3-8]. However, these explanations remain theoretical. The intervention included several components, including meditation, recorded sound, 528 Hz audio, singing bowls, crystal bowls, bells, gongs, and tingshas. It is therefore not possible to determine whether any individual component was responsible for the observed effects. Future studies should use component-specific designs and standardized acoustic parameters to identify which elements contribute most strongly to psychological and physiological outcomes.

Strengths and limitations

The study has several strengths. It used a randomized controlled design, included baseline and post-intervention assessments, evaluated psychological and physiological outcomes, reported confidence intervals and effect sizes, and included baseline-adjusted ANCOVA analyses with Holm adjustment for multiple outcome testing. Outcome assessors responsible for blood pressure measurement and laboratory personnel responsible for salivary cortisol analysis were blinded to group allocation. Intervention procedures and attendance were documented throughout the four-week period. All randomized participants completed post-intervention assessment, and no adverse events were reported.

Several limitations should be considered. Participants were recruited primarily from meditation centers, which may limit generalizability to wider or meditation-naïve populations. The intervention period was limited to four weeks, with no longer-term follow-up. The intervention group attended in-person sessions, whereas the music control group participated remotely through Google Meet. This difference in delivery setting, supervision, audio equipment, room acoustics, and background noise may have influenced outcomes independently of the sound-based intervention. The music control tracks may also have had relaxation-related effects and should not be considered an inert placebo.

Although attendance was recorded for all scheduled center-based sessions, intervention-group home-practice records included both completed and missed entries. The protocol was multicomponent, and the effects of recorded sound, 528 Hz audio, acoustic instruments, supervised meditation, and home practice cannot be separated. Sound-pressure levels were not objectively measured in decibels, and detailed acoustic calibration records were unavailable. In addition, POMS-SF was included as a psychological outcome in the trial registration but was not available in the final analysis dataset. These limitations mean that the findings should be interpreted as exploratory and require confirmation in a larger, fully standardized, protocol-concordant trial with longer follow-up.

## Conclusions

This randomized controlled trial provides preliminary evidence that participation in a structured sound-based meditation program was associated with lower post-intervention anxiety, depression, salivary cortisol, systolic blood pressure, and exploratory diastolic blood pressure than participation in a remotely delivered music control condition, after adjustment for baseline values. The intervention was completed by all randomized participants, and no adverse events were reported. However, the findings should be interpreted as exploratory because of the multicomponent intervention, differences between in-person and remote delivery conditions, incomplete acoustic calibration records, and the absence of POMS-SF data in the final analysis dataset. Larger, fully standardized randomized controlled trials with diverse populations, longer follow-up, and mechanistic assessments are needed.
